# Overview and New Insights Into the Diversity, Evolution, Role, and Regulation of Kisspeptins and Their Receptors in Teleost Fish

**DOI:** 10.3389/fendo.2022.862614

**Published:** 2022-03-22

**Authors:** Bin Wang, Alejandro S. Mechaly, Gustavo M. Somoza

**Affiliations:** ^1^ Key Laboratory of Sustainable Development of Marine Fisheries, Ministry of Agriculture and Rural Affairs, Yellow Sea Fisheries Research Institute, Chinese Academy of Fishery Sciences, Qingdao, China; ^2^ Laboratory for Marine Fisheries and Food Production Processes, Pilot National Laboratory for Marine Science and Technology (Qingdao), Qingdao, China; ^3^ Instituto de Investigaciones en Biodiversidad y Biotecnología (INBIOTEC-CONICET), Mar del Plata, Argentina; ^4^ Fundación para Investigaciones Biológicas Aplicadas (FIBA), Mar del Plata, Argentina; ^5^ Instituto Tecnológico de Chascomús (CONICET-UNSAM), Chascomús, Argentina

**Keywords:** fish, kisspeptin, kisspeptin receptor, reproduction, signaling pathway, gene regulation

## Abstract

In the last two decades, kisspeptin (Kiss) has been identified as an important player in the regulation of reproduction and other physiological functions in vertebrates, including several fish species. To date, two ligands (Kiss1, Kiss2) and three kisspeptin receptors (Kissr1, Kissr2, Kissr3) have been identified in teleosts, likely due to whole-genome duplication and loss of genes that occurred early in teleost evolution. Recent results in zebrafish and medaka mutants have challenged the notion that the kisspeptin system is essential for reproduction in fish, in marked contrast to the situation in mammals. In this context, this review focuses on the role of kisspeptins at three levels of the reproductive, brain-pituitary-gonadal (BPG) axis in fish. In addition, this review compiled information on factors controlling the Kiss/Kissr system, such as photoperiod, temperature, nutritional status, sex steroids, neuropeptides, and others. In this article, we summarize the available information on the molecular diversity and evolution, tissue expression and neuroanatomical distribution, functional significance, signaling pathways, and gene regulation of Kiss and Kissr in teleost fishes. Of particular note are recent advances in understanding flatfish kisspeptin systems, which require further study to reveal their structural and functional diversity.

## Introduction

Eighteen years have passed since the first paper on kisspeptin in a teleost species was published. In that study, the complementary DNA (cDNA) of a kisspeptin receptor (referred to then as *GPR54* and now as *kissr2*) was isolated in the Nile tilapia, *Oreochromis niloticus* (1). The interest in studying the kisspeptin system in fish came from its key role in mammalian reproduction (2–4). A clear example of this is that more than 250 papers have been published to date on kisspeptin and kisspeptin receptors in teleosts, as shown in the Scopus database.

It is now generally accepted that the kisspeptin system in most teleost species consists of two ligands, known as Kiss1 and Kiss2, and two receptors, Kissr2 and Kissr3. However, only one element of this system (either the ligand and/or the receptor) has been detected in Pleuronectiformes, such as *kissr2* in Atlantic halibut, *Hippoglossus hippoglossus* (5); both *kiss2* and *kissr2* in the Senegalese sole, *Solea senegalensis* (6, 7), half-smooth tongue sole, *Cynoglossus semilaevis* (8), and Japanese flounder, *Paralichthys olivaceus* (9). Of note, the European eel, *Anguilla anguilla*, is the only teleost species having two *kiss* genes (*kiss1* and *kiss2*) and three *kissr* types (*kissr1*, *kissr2*, and *kissr3*) reported to date (10).

In recent years, a considerable number of studies have suggested that the kisspeptin system is the “master system” controlling the BPG axis in mammals by exerting its action on gonadotropin-releasing hormone (Gnrh) neurons (3, 11, 12). However, in fish, several studies have considered the Gnrh system as the main system and, the kisspeptin system as a complementary system in controlling fish reproduction (13–15). First, the anatomical association of kisspeptin and Gnrh neurons is not obvious, or almost absent in many teleost species (16–21). Similarly, in zebrafish (*Danio rerio*) and medaka (*Oryzias latipes*) *kiss* and/or *kissr* knockouts display normal reproduction (20, 22, 23). However, it must be considered that, surprisingly, similar results have been obtained with *gnrh* knockouts because, at least in zebrafish, single *gnrh3* mutants and *gnrh3* plus 2 *kiss* gene triple mutants can normally reproduce (23–26). However, in the same species, laser ablation of Gnrh cells at the larval stage resulted in the loss of reproduction in adult fish (27), suggesting that the cellular integrity of Gnrh cells is essential and the Gnrh system is a key and essential player for normal reproduction. Then, it was suggested that the unaltered normal reproductive capacity of mutant fish is compensated by the action of other neuropeptides known to affect gonadotropin secretion (15, 24, 25). In this context, it would be interesting to investigate whether similar mechanisms occur in other teleost species and to clearly determine which peptide(s) are involved in these compensatory mechanisms.

The aim of this review is to examine the entire literature on the kisspeptin system in teleost fishes, with particular emphasis on diversity and evolution, central and peripheral distribution, physiological effects on reproduction, intracellular signaling pathways and regulatory mechanisms.

## Kisspeptin Genes and Peptides

Kisspeptins were initially considered to be members of the RFamide peptide family (10). However, other studies demonstrated that kisspeptins are far from the RF-amide family and were proposed to be members of the Kisspeptin/Galanin/Spexin family (28). Given the low conservation of kisspeptin ligands among fish species, their characterization took longer compared to kisspeptin receptors (7). The first Kiss1 orthologs in fish to be characterized were those of zebrafish, spotted pufferfish (*Tetraodon nigroviridis*), Japanese pufferfish **(** *Fugu rubripes*) and medaka (29, 30). Shortly after, Kiss2 was characterized in zebrafish and medaka (31).

In humans, Kiss1 prepropeptide consists of 145 amino acids (aa) in length, with a major cleavage product of 54 aa (originally named as metastin) and three shorter peptides of 14, 13 and 10 amino acids in length. All these peptides bind to their cognate G protein-coupled receptor today known as kisspeptin receptor (32). It was then demonstrated that the 10 aa peptide was conserved across vertebrates (33), suggesting that it plays an important role in different taxa (34). However, the situation is not as conserved in teleosts. For example, the Kiss1 precursor contains a conserved putative cleavage site six amino acids upstream of the core sequence, suggesting that the mature form of Kiss1 is a pentadecapeptide (17, 35, 36). In addition, the *kiss2* gene produces a mature dodecapeptide in several species (17, 34–36). Moreover, several studies have shown that Kiss1-15 and Kiss2-12 peptides are more effective than Kiss1/2-10 for receptor activation in teleosts (34).

## Kisspeptin Receptor Genes and Proteins

Kisspeptin receptors are membrane receptors that belong to the superfamily of G protein-coupled receptors (GPCRs) (37). These receptors have a highly conserved structure of seven transmembrane domains (TMDs) that has facilitated their cloning and characterization in vertebrates, including teleosts. As mentioned earlier, the first *kissr* to be characterized in fish was found in the Nile tilapia (1). Soon after, several *kissr2* were characterized in other fish species, such as cobia (*Rachycentron canadum*) (38), grey mullet (*Mugil cephalus*) (39), fathead minnow (*Pimephales promelas*) (40) and two flatfish species, Senegalese Sole (6) and Atlantic halibut (5). The zebrafish genome then helped Biran and coworkers (41) to identify for the first time two kisspeptin receptors in a fish species, then named *kiss1ra* and *kiss1rb* and now known as *kissr2* and *kissr3*, according to the nomenclature introduced by Pasquier et al. (10). Since then, two kisspeptin receptors have been discovered in most teleost fish species studied (15, 42, 43). However, not all teleost species have two kisspeptin receptors. For example, only *kissr2* has been found in the three-spined stickleback (*Gasterosteus aculeatus*), fugu (*Takifugu niphobles*), and spotted pufferfish (44). Mechaly and coworkers (5, 6) also failed to detect *kissr3* by PCR in Pleuronectiformes and suggested that *kissr3* may have been lost during evolution of this order (45).

## Evolution of the Kisspeptinergic Systems in Fish

The first phylogenetic studies on the kisspeptin system genes were essentially obtained from cloned and characterized sequences (1, 5–7, 31, 33, 46). However, advances in next-generation sequencing (NGS) technologies and genomics have made available dozens of transcriptomes and genomes from a wide range of teleosts and allowed to re-examine the diversity, origin, and evolution of kisspeptins and their receptors not only in fish but also in other vertebrates (10, 44, 47). For example, sequences related to kisspeptin have been identified from genome databases in the European eel (44) and pejerrey fish, *Odontesthes bonariensis* (36), among others.

**Figure 1:**
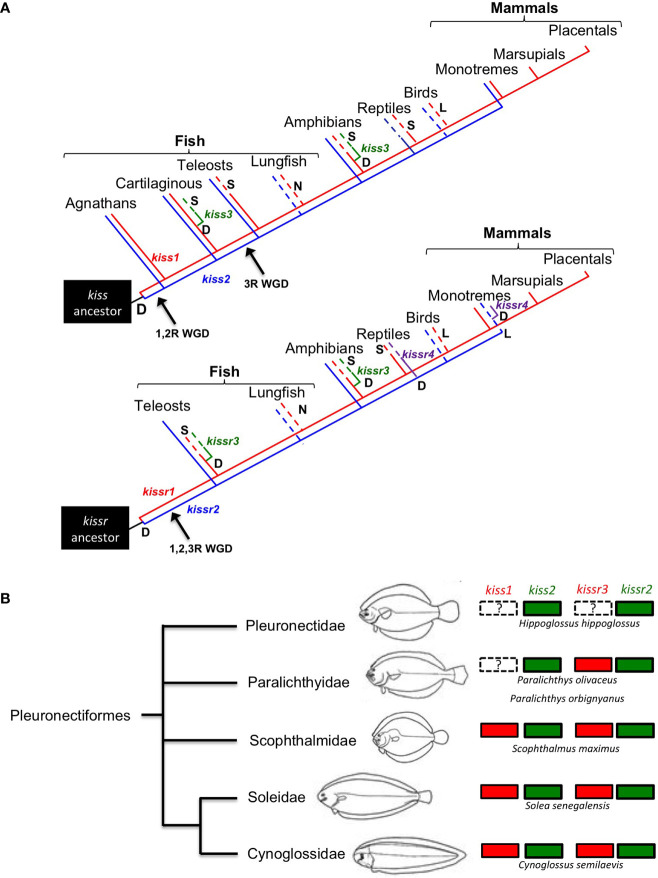
**(A)** *kiss* and *kissr* gene evolution in vertebrates derived from available information. D = gene duplication, L = gene lost, S = gene lost in some species, N = gene not searched. (Modified from 7). **(B)** Consensus tree of flatfish relationships proposed by Chapleau (55), figure modified from Chanet et al. (56).

In teleosts, several studies have performed phylogenetic studies on the nucleotide and/or amino acid sequences of kisspeptins and kisspeptin receptors (10, 44). Since the appearance of this large dataset, more complete phylogenetic studies on the evolution of the kisspeptin systems have been conducted. The situation is similar for flatfishes, and the currently available genomes and/or transcriptomes from half-smooth tongue sole (48), turbot, *Scophthalmus maximus* (49), Senegalese sole (50, 51), common sole, *Solea solea* (50, 51), Japanese flounder (52), black flounder, *Paralichthys orbignyanus* (53), and more recently the available genomes of 11 flatfish species, representing 9 pleuronectiform families (54) have added data to understand kisspeptin phylogeny in teleosts. A summary of all the available information is shown in **Figure 1A**.

Within this framework, another aim of the review is to highlight some relevant aspects of the kisspeptinergic system in flatfishes, as conflicting information on the presence of kisspeptin genes has been reported in this group. One example is the absence of *kiss1* and *kissr3*, as suggested by Mechaly et al. (45) for the Senegalese sole and Atlantic halibut. However, using the current genomic information of both species, we found either complete or partial *kissr3* sequences in Senegalese sole (**Figure 1B**). However, we could not find *kiss1* sequences in the black and Japanese flounders. Both genomes have high sequencing coverage and identified 25,231 protein-coding genes in black flounder (53) and 21,787 protein-coding genes in the case of Japanese flounder (52). However, the absence of *kiss1* annotation in flounder genomes does not necessarily mean that this gene is missing in these species, as “missing” genes can often occur in unassembled reads or contigs (57).

**Figure 2:**
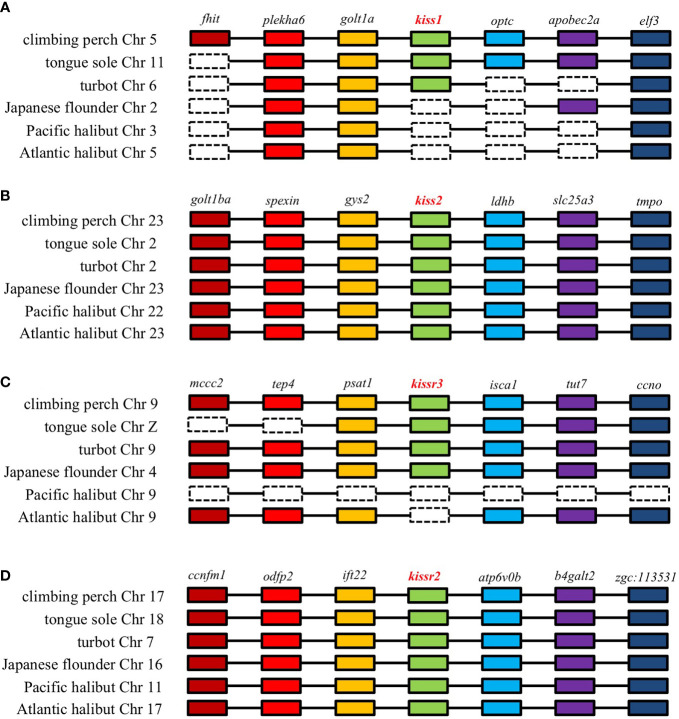
Genomic synteny analysis of kisspeptin **(A, B)** and its receptor **(C, D)** genes in different teleost species.

To make a definitive conclusion about the Kiss1 situation, PCR analysis must be performed in both cases. However, it must be kept in mind that this does not guarantee the detection of the gene, as has already been shown in Senegalese sole and Atlantic halibut (5–7). To test whether *kiss1* and/or *kissr3* have been lost in some pleuronectiform species, a more comprehensive comparative sequence analysis needs to be performed. With this in mind, a syntenic analysis of *kiss* and *kissr* neighboring genes in Pleuronectiformes was performed (**Figure 2**). It is always possible that mutations and/or translocations may have occurred to explain the absence of a particular gene in the genome. For example, a *kiss1*-like transcript was already found in the red seabream, *Pagrus major* (58). A similar situation has also been observed in primates, where a *Kiss2*-like gene was in human, chimpanzee, and gorilla genome databases (59).

## Tissue Expression and Neuroanatomical Distribution of the Kiss/Kissr Systems in Fish

While studies in mammals have shown that kisspeptin has pleiotropic effects (60, 61), the situation in teleosts has not been studied in detail, as most of the studies have focused on the central regulation of reproduction (15, 42, 45). However, kisspeptin transcripts and proteins are widely distributed in various brain areas and tissues of fish, but no clear roles have been associated with these extra brain kisspeptins. These tissues/organs, include the pituitary gland, the spinal cord, the intestine, the gonads, and the liver (62–65). The presence of kisspeptin in blood has been detected in mammals (66). However, to the best of our knowledge there are no available data on kisspeptins levels in fish plasma. This is also the case in flatfishes, where kisspeptin elements have been detected in various tissues, organs and brain areas. As mentioned earlier, only *kiss2* and *kissr2* have been described in all flatfishes studied to date (45). However, to the best of our knowledge, no functions have been assigned to Kiss2 in these tissues/organs. With the genomes available today this situation has changed, as *kiss1* and *kissr3* sequences have been found in several pleuronectiform species, but no functional studies of *kiss1*-*kissr3* tissue expression have been performed to date. In this context, the advent of RNA sequencing technology (RNA-seq) will allow us to clarify this situation. However, future studies are needed to clarify the pleiotropic role of kisspeptins not only in Pleuronectiforms but also in other teleost species.

It is important to mention that alternative splice variants are frequently observed in mammals when analyzing GPCR tissue distribution (67), and spliced variants of kisspeptin have also been detected in several teleosts. For example, the presence of alternative splice variants for *kissr2* and/or *kissr3* was observed in Senegalese sole (6, 45), southern bluefin tuna, *Thunnus maccoyii* (68), yellowtail kingfish, *Seriola lalandi* (68), zebrafish (69), European eel (44) and pejerrey fish (65) through intron retention. Mechaly et al. (6, 45, 65) indicate that truncated and likely non-functional proteins are produced in this manner. In two species, yellowtail kingfish (68) and zebrafish (69) alternative spliced variants were also generated by deletion of exons. For a detailed description of the splicing events detected in kisspeptin receptors in fish see Mechaly et al. (45, 65). In this regards, future studies are needed to evaluate whether truncated proteins are generated and whether this represents regulation by unproductive splicing, as has been described for arginine-serine-rich (SR) splicing factors in several organisms (70). To the best of our knowledge, alternative spliced variants have been detected in a kisspeptin ligand in the specific case of Pleuronectiformes and identified only in the *kiss2* gene of Senegalese sole (7).

## Functional Significance of the Kiss/Kissr System in Fish

In fishes, the functional roles of kisspeptinergic systems in regulating reproduction is not always clear and is sometimes contradictory. For example, Kiss1 has been shown to significantly increase the levels of messengers of luteinizing hormone *b* subunit *(lhb)*, growth hormone (*gh)* and prolactin *(prl)* in goldfish (*Carassius auratus*) pituitary cells (71). However, in European eel, four different kisspeptin analogues (Kiss1-10, Kiss1-15, Kiss2-10 and Kiss2-12) are able to specifically inhibit *lhb* expression in a dose-dependent manner without affecting follicle-stimulating hormone *b* (*fshb)* mRNA levels when acting on pituitary cells (72). But, as mentioned earlier, *kiss*/*kissr* mutated fish showed almost normal fertility and gonadal maturation, suggesting that kisspeptin systems are not strictly required for reproduction, at least in some teleosts (20, 22) or compensatory mechanisms may take over the role of kisspeptins in reproduction (20, 22, 43, 73, 74).

Moreover, connection of kisspeptin nerve terminals and Gnrh cells is not really clear in all teleost species. For example, in the Nile tilapia, a kisspeptin receptor has been shown to be expressed in Ghrh cells (1), and a small number of Gnrh neurons receive kisspeptin innervation in zebrafish (16, 21), striped bass (*Morone saxatilis*) (17) and the cichlid *Astatotilapia burtoni* (75). Conversely, in medaka (19) and European sea bass (*Dicentrarchus labrax*) (18), the presence of kisspeptin receptors on Gnrh neurons could not be detected. However, in zebrafish, Kiss2 nerve terminals reach the pituitary gland (76) and Kiss2 cell bodies and fiber-like projections are found in the *proximal pars distalis* (PPD) with a distribution like Gnrh3 nerve terminals (77), supporting the possibility of an intrapituitary kisspeptinergic regulation of pituitary function. Thus, the physiological significance and functions of kisspeptin in fish reproduction remain controversial. **Table 1** summarizes the physiological effects of kisspeptins in teleosts fish.

**Table 1:** Summary of physiological effects of kisspeptins in fish.

Species (Common names)	Kisspeptin types	Peptide sequences	Physiological actions	References
*Carassius auratus* (Goldfish)	Kiss1-10	YNLNSFGLRY-NH2	Stimulation of pituitary LH, GH and PRL release and synthesis *in vitro*	Yang et al. (71)
			Stimulation of pituitary SLa release *in vitro*	Jiang et al. (78)
			Increase of plasma LH levels *in vivo*	Li et al. (33)
			Stimulation of brain and ovary *kiss1* synthesis *in vivo*	Valipour et al. (79)
	Kiss2-10	FNYNPFGLRF-NH2	No effect on LH release both *in vivo* and *in vitro*	Li et al. (33)
			Stimulation of pituitary *fshb* and *lhb* synthesis *in vivo*	Valipour et al. (80)
			Increase of plasma 17*b*-estradiol levels *in vivo*	Valipour et al. (80)
*Danio rerio* (Zebrafish)	Kiss1-10	YNLNSFGLRY-NH2	No effect on brain *gnrh2* and *gnrh3* synthesis as well as pituitary *fshb*, *lhb*, *gh1* and *prl* synthesis *in vivo*	Kitahashi et al. (31)
	Kiss2-10	FNYNPFGLRF-NH2	Stimulation of pituitary *fshb* and *lhb* synthesis *in vivo*	Kitahashi et al. (31)
			Stimulation of pituitary *fshb, lhb* and *prl1* synthesis in females *in vitro*	Song et al. (77)
*Morone saxatilis* × *Morone chrysopshy* (Hybrid bass)	Kiss1-15	QDVSSYNLNSFGLRY-NH2	Increase of plasma LH levels at gonadal recrudescence *in vivo*	Zmora et al. (17)
			Stimulation of brain *gnrh1* synthesis at prepuberty *in vivo*	Zmora et al. (17)
			Inhibition of brain *kissr2* synthesis at recrudescence *in vivo*	Zmora et al. (17)
	Kiss2-12	SKFNFNPFGLRF-NH2	Increase of plasma LH levels at prepuberty and gonadal recrudescence *in vivo*	Zmora et al. (17)
			Stimulation of brain *kissr2* and *gnrh1* synthesis at prepuberty *in vivo*	Zmora et al. (17)
			Inhibition of brain *kissr2* and *gnrh1* synthesis at gonadal recrudescence *in vivo*	Zmora et al. (17)
*Morone saxatilis* (Striped bass)	Kiss1-15	QDVSSYNLNSFGLRY-NH2	Inhibition of brain *gnrh1* and *gnrh2* synthesis *in vivo*	Zmora et al. (81)
			Inhibition of pituitary *gnrh1r* synthesis *in vivo*	Zmora et al. (81)
			Stimulation of pituitary *fshb* synthesis *in vivo*	Zmora et al. (81)
			Increase of oocyte diameter *in vivo*	Zmora et al. (81)
			Stimulation of pituitary *fshb* synthesis *in vitro*	Zmora et al. (82)
			Inhibition of pituitary *lhb* synthesis *in vitro*	Zmora et al. (82)
			Increase of FSH levels *in vitro*	Zmora et al. (82)
	Kiss2-12	SKFNFNPFGLRF-NH2	Inhibition of brain *gnrh1*, *gnrh2* and *gnrh3* synthesis *in vivo*	Zmora et al. (81)
			Inhibition of pituitary *gnrh1r* synthesis *in vivo*	Zmora et al. (81)
			Decrease of plasma LH and FSH levels *in vivo*	Zmora et al. (81)
			Stimulation of brain *gnrh1* synthesis *in vitro*	Zmora et al. (82)
			Stimulation of pituitary *fshb* synthesis *in vitro*	Zmora et al. (82)
			Increase of FSH and LH levels *in vitro*	Zmora et al. (82)
*Dicentrarchus labrax* (European sea bass)	Kiss1-10 Kiss1-15	YNLNSFGLRY- NH2 QDVSSYNLNSFGLRY- NH2	Increase of plasma LH levels *in vivo*	Felip et al. (46)
			Stimulation of *kissr2* synthesis in forebrain-midbrain *in vivo*	Espigares et al. (83)
			Inhibition of *gnrh1* and *gnrh2* synthesis in forebrain-midbrain *in vivo*	Espigares et al. (83)
			Increase of hypothalamic and pituitary GnRH1 content *in vivo*	Espigares et al. (83)
			Increase of plasma LH levels *in vivo*	Espigares et al. (83)
	Kiss2-10 Kiss2-12	FNFNPFGLRF-NH2 SKFNFNPFGLRF-NH2	Increase of plasma LH and FSH levels *in vivo*	Felip et al. (46)
			Stimulation of *kissr2* synthesis in forebrain-midbrain *in vivo*	Espigares et al. (83)
			Inhibition of *gnrh1* and *gnrh2* synthesis in forebrain-midbrain *in vivo*	Espigares et al. (83)
			Increase of hypothalamic GnRH1 content *in vivo*	Espigares et al. (83)
			Inhibition of pituitary *gnrhr*-*II*-*1a* synthesis *in vivo*	Espigares et al. (83)
			Increase of plasma LH, T and 11-KT levels *in vivo*	Espigares et al. (83)
			Increase of sperm motility parameters *in vivo*	Espigares et al. (83)
			Stimulation of pituitary LH and FSH release *in vitro*	Espigares et al. (84)
*Scomber japonicus* (Chub mackerel)	Kiss1-10	QDMSSYNFNSFGLRY-NH2	Inhibition of pituitary *lhb* synthesis in sexually immature adult females *in vivo*	Selvaraj et al. (85)
			Increase of plasma 11-KT levels in sexually immature adult males and E2 levels in females *in vivo*	Selvaraj et al. (85)
			Induction of spermiation and vitellogenic onset *in vivo*	Selvaraj et al. (85)
			Increase of plasma 11-KT and E2 levels in pre-pubertal males *in vivo*	Selvaraj et al. (86)
			Acceleration of spermatogenesis in pre-pubertal males *in vivo*	Selvaraj et al. (86)
	Kiss2-12	SNFNFNPFGLRF-NH2	Inhibition of brain *gnrh1* synthesis in sexually immature adult females *in vivo*	Ohga et al. (87)
			Stimulation of pituitary *fshb* and *lhb* synthesis in both sexes *in vivo*	Ohga et al. (87)
			Stimulation of brain *gnrh1* synthesis in sexually immature adult females *in vivo*	Selvaraj et al. (85)
			Increase of spermatocytes numbers in pre-pubertal males *in vivo*	Selvaraj et al. (86)
*Seriola lalandi* (Yellowtail kingfish)	Kiss1-10	YNLNSFGLRY-NH2	Stimulation of pituitary *kissr2* synthesis during the non-breeding season *in vivo*	Nocillado et al. (88)
			Stimulation of pituitary *fshb* synthesis during the breeding season *in vivo*	Nocillado et al. (88)
			Stimulation of pituitary *fshb* and *lhb* synthesis during the non-breeding season *in vivo*	Nocillado et al. (88)
			Stimulation of gonadal development regardless of the season *in vivo*	Nocillado et al. (88)
	Kiss2-10	FNFNPFGLRF-NH2	Stimulation of gonadal development during the non-breeding season *in vivo*	Nocillado et al. (88)
			Inhibition of brain *kissr2_v1* and *kissr2_v5* synthesis in pre-pubertal males *in vivo*	Nocillado et al. (68)
			Stimulation of *kissr2_v4* synthesis in pre-pubertal males *in vivo*	Nocillado et al. (68)
			Increase of plasma E2 levels in pre-pubertal females *in vivo*	Nocillado et al. (68)
*Anguilla anguilla* (European eel)	Kiss1-10 Kiss1-15	YNWNSFGLRY-NH2 ENFSSYNWNSFGLRY-NH2	Inhibition of pituitary *lhb* and *gnrhr*-2 synthesis *in vitro*	Pasquier et al. (72)
	Kiss2-10 Kiss2-12	FNRNPFGLRF-NH2 SKFNRNPFGLRF-NH2	Inhibition of pituitary *lhb* and *gnrhr*-2 synthesis *in vitro*	Pasquier et al. (72)
*Cynoglossus semilaevis* (Half-smooth tongue sole)	Kiss2-10	FNFNPFGLRF-NH2	Stimulation of hypothalamic *kiss2* and *lpxrfa* synthesis *in vitro*	Wang et al. (89)
			Inhibition of hypothalamic *kissr2* and *lpxrfa-r* synthesis *in vitro*	Wang et al. (89)
			Stimulation of pituitary *fshb* and *gtha* synthesis *in vitro*	Wang et al. (90)
*Epinephelus coioides* (Orange-spotted grouper)	Kiss2-10	FNFNPFGLRF-NH2	Stimulation of hypothalamic *gnrh1* synthesis *in vivo*	Shi et al. (91)
			Stimulation of pituitary *fshb* synthesis *in vivo*	Shi et al. (91)
*Oreochromis niloticus* (Nile tilapia)	Kiss2-10	FNYNPLSLRF-NH2	Stimulation of brain *gnrh1*, *fshb* and *lhb* synthesis *in vivo*	Park et al. (92)
			Increase of plasma 11-KT levels in males and E2 levels in females *in vivo*	Park et al. (92)
			Acceleration of spermatogenesis *in vivo*	Park et al. (92)
*Hippocampus erectus* (Lined seahorse)	Kiss2-10	FNVNPFGLRF-NH2	Stimulation of pituitary *fshb* and *lhb* synthesis *in vivo*	Zhang et al. (93)
			Increase of plasma testosterone levels *in vivo*	Zhang et al. (93)
*Solea senegalensis* (Senegalese sole)	Kiss2-10	FNFNPFGLRF-NH2	Increase of plasma FSH and LH levels *in vivo*	Oliveira et al. (94)
			Increase of plasma testosterone levels *in vivo*	Oliveira et al. (94)
*Heteropneustes fossilis* (Tinging catfish)	Kiss1-10	YNWNSFGLRY-NH2	Stimulation of hypothalamic, pituitary and ovarian *gnrh1* and *gnrh2 in vivo*	Chaube et al. (95)
			Stimulation of pituitary *fshb* and *lhb* synthesis *in vivo*	Chaube et al. (95)
			Increase of plasma and ovarian E2, progesterone and 17,20b-dihydoxy-4-pregnen-3-one levels	Chaube et al. (95)
	Kiss2-10	FNFNPFGLRF-NH2	Stimulation of hypothalamic, pituitary and ovarian *gnrh1* and *gnrh2 in vivo*	Chaube et al. (95)
			Stimulation of pituitary *fshb* and *lhb* synthesis *in vivo*	Chaube et al. (95)
			Increase of plasma and ovarian E2, progesterone and 17,20b-dihydoxy-4-pregnen-3-one levels	Chaube et al. (95)
*Micropterus salmoides* (Largemouth bass)	Kiss2-10	FNFNPFGLRF-NH2	Stimulation of brain *gnrh3* and *kissr2* synthesis *in vivo*	Li et al. (96)
			Stimulation of pituitary *fshb* and *lhb* synthesis *in vivo*	Li et al. (96)
			Stimulation of ovarian *er2* and testicular *ar* synthesis *in vivo*	Li et al. (96)
			Increase of plasma 17*b*-estradiol and testosterone levels *in vivo*	Li et al. (96)
			Acceleration of vitellogenesis and spermatogenesis *in vivo*	Li et al. (96)

## Kisspeptins’ Actions at Brain Level

The biological effects of kisspeptin on Gnrh neurons have been demonstrated in several teleost species at different levels. Kiss1 stimulates the electrical activity of terminal nerve-Gnrh3 neurons in adult medaka (97). Kiss1 also stimulates the electrical activity of the preoptic area (POA) and hypothalamic Gnrh3 neurons in adult zebrafish, while Kiss2 inhibits their neuronal activity (98). In the orange-spotted grouper, *Epinephelus coioides*, intraperitoneal (ip) injection of Kiss2 leads to upregulation of hypothalamic expression of *gnrh1* (91). Similarly, Kiss2, but not Kiss1, significantly stimulates *gnrh1* expression in striped bass brain slices (82). Stimulatory effects of Kiss2 on *gnrh1* expression in the brain and hypothalamus are also observed in the black porgy, *Acanthopagrus schlegelii* (99), Nile tilapia (92), and Japanese flounder (100). In hybrid bass, a differential and gonadal stage-dependent role of kisspeptins on *gnrh1* expression in the brain was observed; both Kiss1 and Kiss2 increase *gnrh1* expression in pre-pubertal fish, while Kiss2 reduces *gnrh1* expression in gonadal recrudescencing fish (17).

Chronic administration of Kiss1 and Kiss2 leads to a decrease in *gnrh1*, *gnrh2*, and/or *gnrh3* transcript levels in the brain of female striped bass (81). An inhibitory effect of Kiss1 and Kiss2 on *gnrh1* and *gnrh2* expression in the forebrain and midbrain is also found in male European sea bass (83). On the other hand, Kiss2 does not alter *gnrh2* and *gnrh3* mRNA expression in the hypothalamus of the half-smooth tongue sole *in vitro* (89). Likewise, injection with Kiss1 and/or Kiss2 peptides induce no significant differences in *gnrh* mRNA levels in other teleosts, such as zebrafish brain *gnrh2* and *gnrh3* (31), hybrid bass brain *gnrh2* and *gnrh3* (17), orange-spotted grouper hypothalamic *gnrh3* (91), yellowtail kingfish brain and hypothalamic *gnrh1* (88), lined seahorse hypothalamic *gnrh3* (93), European sea bass hypothalamic *gnrh1* and forebrain-midbrain *gnrh3* (83). However, Kiss2 has both stimulatory and inhibitory effects on *gnrh1* mRNA levels in the brain of female chub mackerel (*Scomber japonicus*), depending on the mode of administration. Subcutaneous and slow release of Kiss2 increases *gnrh1* expression (86), whereas intracerebroventricular (icv) administration of Kiss2 suppresses *gnrh1* expression (87). Taken together, these data suggest that the mode of actions of Kiss1 and Kiss2 on Gnrh neurons are different among fish species and depend not only on gonadal status but also on the way of administration.

On the other hand, the LPXRFa system (the piscine ortholog of gonadotropin-inhibitory hormone, Gnih) is also a target for the central effects of kisspeptin in fish. In hypothalamic explants of half-smooth tongue sole, Kiss2 exerts a stimulatory effect on *lpxra* transcript levels, while apparently reducing *lpxrfa-r* mRNA levels (89). To our knowledge, this is the first evidence for the involvement of kisspeptin in the LPXRFa system in any fish species investigated so far. In addition, autoregulation of the kisspeptin system has been observed in several teleosts. For details, see the section on neuropeptides (see below).

## Kisspeptins´ Actions on the Pituitary

The physiological roles of both Kiss1 and Kiss2 do not appear to follow a common pattern in teleosts. Previous *in vitro* studies indicate a direct stimulatory effect of kisspeptin on gonadotropins in different species. For example, Kiss1 significantly triggers Lh release from primary pituitary cell cultures of goldfish (71, 101), and Kiss2 has a stimulatory effect on both Lh and Fsh release in pituitary cells of European sea bass (84). In striped bass, both Kiss1 and Kiss2 stimulate Fsh release *in vitro*, whereas only Kiss2 is able to exert a stimulatory effect on Lh release (82). Moreover, ip injection of Kiss1, but not Kiss2, significantly increases serum Lh levels in goldfish (33). An increase in plasma Lh and Fsh levels is observed in European sea bass after injection of both Kiss1 and Kiss2 (46, 83). However, Kiss2 is more effective than Kiss1 in triggering gonadotropin secretion in this species (46). Similarly, intramuscular injection of Kiss2 stimulates secretion of Fsh and Lh in Senegalese sole of both sexes (94). Moreover, a differential and gonadal stage-dependent roles of kisspeptin on Lh release was observed in hybrid bass: Kiss1 increases plasma LH levels during gonadal recrudescence *in vivo*, whereas Kiss2 stimulates the release of LH during at pre-puberty and gonadal recrudescence (17).

In addition, injection of Kiss2 triggers an increase in pituitary *fshb* and *lhb* mRNA expression in zebrafish (31) and chub mackerel (87). Treatment of orange-spotted grouper with Kiss2 results in an increase in *fshb* mRNA abundance *in vivo* (91). Moreover, half-smooth tongue sole Kiss2 apparently induces an increase in *gtha* and *fshb* mRNA levels, without affecting *lhb* mRNA transcripts *in vitro* (90). In zebrafish, Kiss2 significantly stimulates *fshb* and *lhb* expression in the female pituitary gland *in vitro* (77).

However, other teleost studies have reported some inhibitory effects of kisspeptins on gonadotropins. For example, chronic treatment with Kiss2 results in a decrease in plasma Lh and Fsh levels *in vivo* in striped bass (81). Both heterologous and homologous kisspeptin peptides inhibit *lhb* mRNA levels *in vitro*, without affecting *fshb* expression in European eel (72, 102). An inhibitory effect of Kiss1 on *lhb* expression is also observed in striped bass (82) and female chub mackerel (85). However, no effect of Kiss1 treatment on the relative abundances of *lhb* and *fshb* is observed in zebrafish (31) and chub mackerel (86, 87). Kiss2 also dose not alter *lhb* and *fshb* mRNA levels in yellowtail kingfish (88), chub mackerel (85), striped bass (81) and European sea bass (84).

In addition to the effects on gonadotropins, kisspeptins have also been shown to be involved in regulating the synthesis and/or release of other pituitary hormones in fish. Kiss1 in goldfish directly stimulates the secretion of Prl and Gh as well as gene expression *in vitro* (71). Similarly, Kiss1 enhances the release of somatolactin-a (Sla) in goldfish pituitary cells (78). Kiss2 in zebrafish significantly stimulates the expression of *prl1* in the female pituitary *in vitro* without affecting the mRNA levels of *prl2*, pro-opiomelanocortin-a (*pomca*) and *pomcb* (77). At the pituitary level, injection of Kiss1, but not Kiss2, significantly increases pituitary levels of Gnrh1 in European sea bass (83). In addition, an inhibitory effect of kisspeptin on *gnrhr* expression is observed in European eel (72), European sea bass (83) and striped bass (81). As mentioned earlier, it is also important to emphasize that unidentified Kiss2 cells and projections are found in the PPD, as well as the distribution of Gnrh3 fibers (77), suggesting the possibility of a paracrine/autocrine intrapituitary kisspeptinergic system.

## Kisspeptins´ Actions on the Gonads

To date, there are a few reports on the effects or functions of kisspeptins at the gonadal level in teleosts. An initial study in yellowtail kingfish showed that chronic treatment with Kiss1 and Kiss2 could stimulate gonadal development in prepubertal males (103). Further studies in the same species showed that Kiss1 is more effective in stimulating gonadal development during the breeding season, while the effects of Kiss2 is more pronounced during the nonbreeding season (88). Kiss1 is also able to accelerate puberty onset in juvenile male white bass (*Morone chrysops*) (104). Plasma levels of 11-ketotestosterone (11-KT) and 17b-estradiol are increased, and spermatogenesis and the onset of vitellogenesis are observed in sexually immature adult chub mackerel over 6–7 weeks following subcutaneous implantation of Kiss1, but not Kiss2 (85). Furthermore, subcutaneous injection of Kiss1 also accelerates spermatogenesis in prepubertal male chub mackerel (86).

On the other hand, only Kiss2 stimulates plasma levels of testosterone (T) and 11-KT in male European sea bass, causing an increase in cumulative milt, sperm density and sperm motility parameters (83). Similarly, plasma levels of 11-KT in males and E_2_ in females are significantly increased in immature Nile tilapia treated with Kiss2, and Kiss2 apparently accelerates the process of spermatogenesis (92). Recently, Kiss2 was shown to stimulate T secretion in both sexes of Senegalese sole (94). All these data suggest an effect on the gonads, probably mediated by gonadotropins, but a direct effect of kisspeptins on the gonads have been less considered.

It is now known that there is intra-gonadal expression of kisspeptins and kisspeptin receptors in fish gonads, suggesting a local action on fish gonads (36, 42, 62, 63, 105–110). In this context, the intra-gonadal roles of kisspeptin in fish are poorly understood. For example, Kiss1 was recently detected in the gonads of Asian catfish (*Clarias batrachus*) and it was suggested that it could locally regulate gonadal steroidogenesis (111, 112). In addition, Chaube et al. (95) found that kisspeptins in female stinging catfish, *Heteropneustes fossilis*, act not only at the brain or pituitary level but also on the ovary to stimulate ovarian maturation and ovulation, demonstrating the potential of these peptides for aquaculture.

Taken together, these results suggest that kisspeptins may regulate the reproductive axis by acting not only at the brain and pituitary level but also at the gonadal level in teleost species.

## Other Physiological Roles

Less explored and beyond the control of reproduction, kisspeptins are involved in other physiological processes in fish. For example, mammalian kisspeptin increases the expression of pituitary *gh*, *sl*, melatonin receptor (*mt*), and hepatic insulin growth factor-1 (*igf-1)*, along with higher levels of plasma Gh, Igf-1, and melatonin in the cinnamon clownfish (*Amphiprion melanopus*), suggesting a role in controlling growth in this species (113). On the other hand, intracranial administration of Kiss1 suppresses the fear response elicited by an alarm substance (AS) in zebrafish, representing a unique role for the Kiss1 system in the brain of teleosts (114). Further studies in the same species showed that Kiss1 reduces the AS-triggered fear response *via* serotonin receptors (115).

However, whether and how kisspeptins are involved in the control of food intake and energy balance in fish remains unknown and represents a promising area for future research, as nutritional status has a profound effect on *kiss*/*kissr* gene expression in some teleosts (7, 65, 116).

## Signaling Pathways Activated by the Kiss/Kissr Systems in Fish

Despite the importance of studying the involvement of kisspeptins in the regulation of reproduction in fish, the detailed intracellular signaling pathways mediating the effects of the Kiss/Kissr systems have not been fully elucidated (2, 10, 117). In these studies, mainly heterologous mammalian cell lines transfected with fish cognate receptors were used together with cAMP-responsive element-dependent luciferase (CRE-luc) or serum responsive element-dependent luciferase (SRE-luc) reporter assays to investigate the possible involvement of the protein kinase A (PKA) or protein kinase C (PKC) pathways, respectively (118, 119).

Analysis of zebrafish Kissr3 signal transduction in COS-7 cells reveals a clear stimulation of CRE-luc activity and SRE-luc activity by Kiss1, suggesting that zebrafish Kissr3 signal can be transduced *via* both PKA and PKC pathways, whereas Kissr2 transduces its activity through the PKC pathway (41). Similarly, in zebrafish, both Kiss1 and Kiss2 induce a concentration-dependent increase in SRE-luc activity in CV-1 cells, CHO-K1 cells, and HEK293 cells expressing their cognate receptors (35, 69). In chub mackerel and medaka, however, Kissr3 activity is transduced *via* the PKC pathway, whereas Kissr2 signaling is transduced *via* both the PKA and PKC pathways (19, 120). Similar results are also observed for Kissr3 signaling in Pacific bluefin tuna, *Thunnus orientalis* and Japanese Spanish mackerel, *Scomberomorus niphonius* (121).

On the other hand, in striped bass, both Kissr2 and Kissr3 are signaling through the PKC pathway rather than the PKA pathway (82). Interestingly, in goldfish and European sea bass Kissr2 and Kissr3 signals can be transduced *via* both the PKA and PKC pathways (33, 122). To date, only the Kissr2 type has been identified in orange-spotted grouper, half-smooth tongue sole, yellowtail kingfish and Southern bluefin tuna, and differential activation of the signal transduction pathways has been demonstrated. In the case of orange-spotted grouper, Kiss2 activates the PKC pathway, but not the PKA pathway (91). However, in the other three species, Kissr2 signaling is shown to be transduced *via* both the PKC and PKA pathways (68, 123). In addition, blockade of the PKC and PKA pathways by specific inhibitors significantly reduces the stimulatory effects induced by half-smooth tongue sole Kiss2, further confirming the participation of these two signaling pathways in the action of Kissr2 (123).

It is worth noting that the coexistence of two Kiss/Kissr systems in a single fish species indicates differential ligand selectivity for the two cognate receptors. In general, Kissr2 and Kissr3 exhibit higher affinity for Kiss2 and Kiss1, respectively, as observed in zebrafish (69), chub mackerel (120), medaka (19), and European sea bass (122). However, in goldfish, Kissr3 is more efficiently activated by Kiss2, whereas Kissr2 is preferentially activated by Kiss1 (33). In striped bass, Kissr3 is activated almost equally by Kiss1 and Kiss2, and Kissr2 is activated more efficiently by Kiss2 than by Kiss1 (82). It is noteworthy that the longer ligand forms show a stronger efficacy in activating the receptors than the core decapeptide (35, 82, 120–122).

In addition, the possible involvement of intracellular Ca^2+^ was also evaluated among post-receptor signaling events evoked by kisspeptin, showing that all European eel kisspeptin forms are able to increase intracellular Ca^2+^ in CHO-K1 cells stably transfected with the rat Kissr1 (72). It should be noted that the European eel is the only teleost species that possesses three different kisspeptin receptors (Kissr1, Kissr2, and Kissr3) that have been studied to date. However, there is no information on the signaling pathways triggered by homologous Kiss peptides across each Kissr type of eel (10, 72). On the other hand, other studies were performed using primary cultured pituitary cells to investigate the molecular mechanisms of the effects of the Kiss/Kissr system on target cells. Consistent with the results obtained with the heterologous systems mentioned above, goldfish Kiss1 may act directly at the pituitary level to increase SLa release *via* the PKA and PKC pathways and subsequent activation of Ca^2+^-dependent cascades (78). Goldfish Kiss1 also directly stimulates the secretion of Lh and Gh from primary cultures of pituitary cells in a Ca^2+^-dependent manner (101). Moreover, Kiss2 is shown to increase phosphorylation levels of ERK and Akt in female pituitary explants in zebrafish (77).

**Figure 3:**
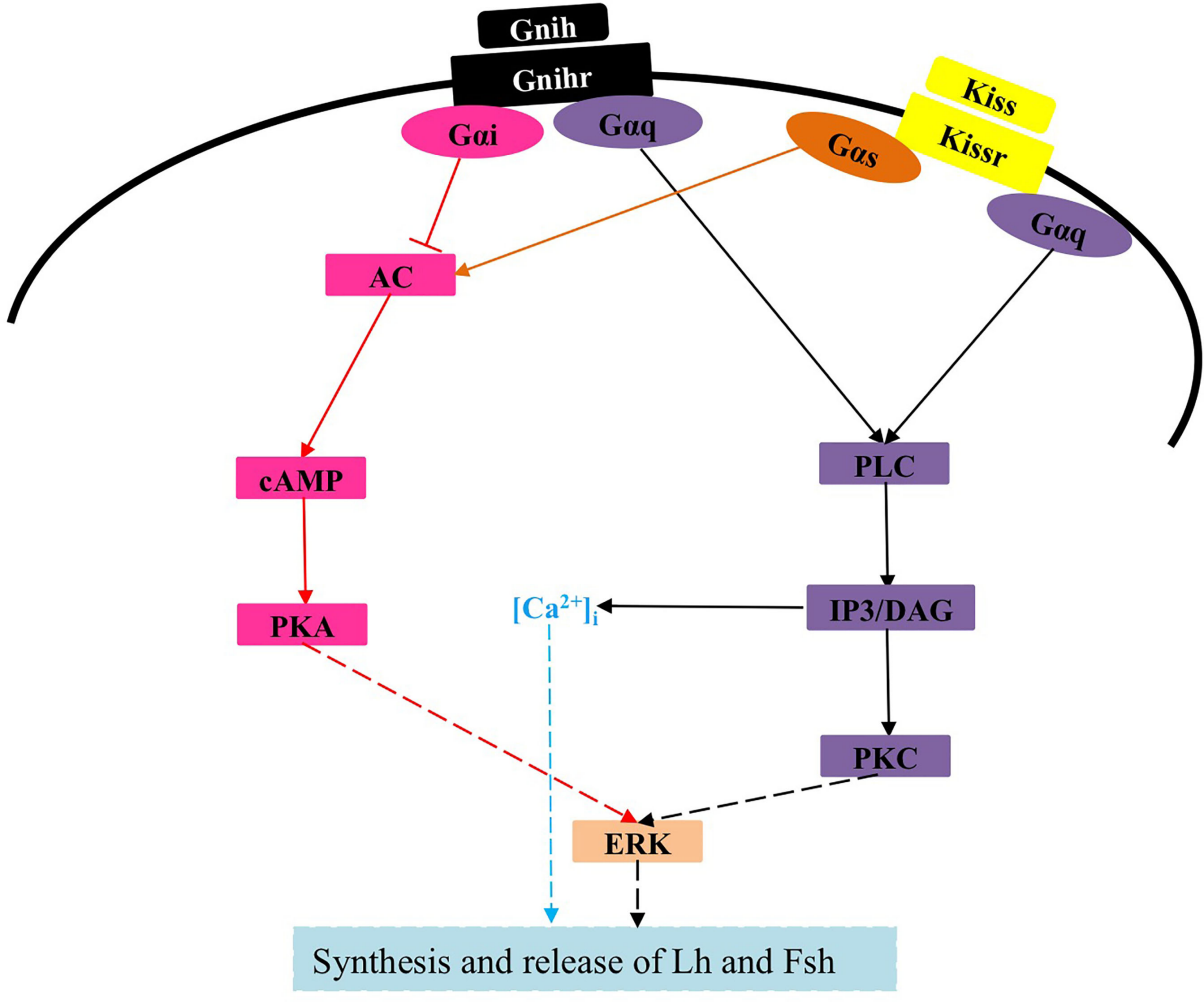
Signaling pathway of Kissr and possible interaction with Gnih in teleosts. The solid lines represent confirmed effects, whereas the dashed lines indicate very limited evidence or possible pathways and interactions that merit further investigation. Kiss, kisspeptin; Kissr, Kiss receptor; Gnih, gonadotropin-inhibitory hormone; Gnihr, Gnih receptor; Gαq, Gαs and Gαi, heterotrimeric G proteins; PLC, phospholipase C; IP3, inositol 1,4,5-trisphosphate; DAG, diacylglycerol; PKC, protein kinase C; ERK, extracellular signal-regulated kinase; AC, adenylyl cyclase; PKA, protein kinase A; Lh, luteinizing hormone; Fsh, follicle-stimulating hormone.

Currently, there is limited information on the interaction between kisspeptins and other neuroendocrine factors in cell signaling (124). In zebrafish, none of the three LPXRFa peptides (LPXRFa-1, LPXRFa-2, and LPXRFa-3) alters SRE-luc activity in COS-7 cells transfected with any of the three cognate LPXRFa receptors (LPXRFa-R1, LPXRFa-R2, and LPXRFa-R3), however, both LPXRFa-2 and LPXRFa-3 exert an inhibitory effect on Kiss2 activation of Kissr2, which involves the PKC pathway (125). Moreover, LPXRFa-2, but not LPXRFa-3, also inhibits Kiss1 activation of Kissr3, which involves the PKC pathway (125). Similarly, half-smooth tongue sole LPXRFa-1 and LPXRFa-2 can also antagonize the action of Kissr2 by inhibiting the PKC pathway (90). Because half-smooth tongue sole LPXRFa-R is coupled to Gαi protein (126), whereas its Kissr2 is coupled to Gαs protein (123), thus LPXRFa-2 also exerts an inhibitory effect on Kissr2 signaling involving the PKA pathway (123). Of note, Kissr3, LPXRFa-R2, and LPXRFa-R3 all transduce their activity through the PKA pathway in zebrafish (41, 125), but no comparative studies have been conducted. Given that activation of Kissr1 in mammals is coupled to multiple signals (10, 12, 127), further studies are needed to investigate previously unknown intracellular mechanisms by which kisspeptin exerts its physiological functions in teleosts, as well as possible interactions of kisspeptins with other factors (**Figure 3**).

## Regulation of the Kiss/Kissr Systems in Fish

### Photoperiod (Melatonin)

In mammals, kisspeptin is recognized as a mediator of photoperiodic control of reproduction, and the effects of photoperiod are mainly by melatonin produced in the pineal gland during the night (128, 129). Nevertheless, studies on the effects of photoperiod on the kisspeptin system are still scarce and in some way contradictories in teleosts. For example, an initial study in Nile tilapia showed that continuous illumination reduces brain *kissr2* expression levels, suggesting a possible link between environmental stimuli and the kisspeptin system (130). In contrast, continuous light increases hypothalamic *kissr2* expression levels in Atlantic salmon (64). On the other hand, there is no clear relationship between *kiss2*/*kissr2* expression and photoperiod in Atlantic cod (131).

In medaka, a long-day (LD) breeder, the number of *kiss1* neurons located in the *Nucleus ventral tuberis* (NVT) in the LD condition is larger than that in the short-day (SD) condition, whereas the *kiss2* neurons located in the *Nucleus recessus lateralis* (NRL) are not altered (29, 132). On the contrary, *kiss2*, but not *kiss1*, transcript levels in the brain of striped bass/white bass hybrid, a SD spawner, increase in the SD regime compared to the LD regime (133). In zebrafish, a LD breeder, constant darkness increases brain melatonin concentrations, and melatonin stimulates *kiss1* and *kiss2* gene expression in the brain (134, 135). Similarly, melatonin elicits a significant increase in *kiss1*, *kiss2* and *kissr2* mRNA abundance in the hypothalamus of male European sea bass (136), while an inhibitory effect of melatonin on *kiss1* and *kiss2* mRNA levels is observed in the dorsal brain of male European sea bass (136) and in the whole brain of female sapphire devil, *Chrysiptera cyanea* (137). Furthermore, continuous light results in the loss of forebrain-midbrain *kiss1*/*kissr3* seasonal rhythms in male European sea bass, which apparently prevents further normal testicular development (138). Taken together, these results indicate that the effects of photoperiod mediated by melatonin can regulate the *kiss*/*kissr* systems. This appeared to be species- and tissue-specific, and the mechanisms of action remain to be studied in detail in fish.

### Temperature

Temperature, especially in ectothermic vertebrates is one of the most important environmental factors regulating reproduction. However, the mechanism by which temperature affects reproduction remains unclear in teleosts. Preliminary evidence has indicated that kisspeptin systems may be involved in mediating the effects of temperature on reproduction. For example, in sexually mature male zebrafish temperature differentially modulated gene expression of the two kisspeptin systems (139). A low temperature of 15°C, but not a high temperature of 35°C, significantly increases *kiss1* mRNA levels in the whole brain, as well as *kissr3* mRNA levels in the habenula and the brain region containing nucleus of the medial longitudinal fascicle, the oculomotor nucleus, and the interpeduncular nucleus. However, *kiss2* mRNA levels in the whole brain and *kissr2* mRNA levels in the caudal zone of the periventricular hypothalamus and the *posterior tuberal nucleus* is significantly decreased when exposed to both low and high temperatures. Interestingly, *kissr2* mRNA levels in the nucleus of the medial longitudinal fascicle, the oculomotor nucleus, and interpeduncular nucleus show an increase when animals were exposed to low temperatures compared with the normal rearing temperature for this species, 27°C. These results suggest that the *kiss1*/*kissr3* system is activated by low temperatures, whereas the *kiss2*/*kissr2* system is inhibited by both low and high temperatures, suggesting that these two kisspeptin systems may be involved in different aspects of zebrafish physiology (139).

Similarly, an inhibitory effect on the expression of *kiss2* and *kissr2* genes is also observed in the diencephalon/midbrain of mature male grass puffer, that spawns on the beach in semilunar cycles during spring tide in early summer, when exposed to both low and high temperatures (140). Notably, although brain melatonin concentrations are significantly increased at high temperatures, high temperatures do not affect *kiss2* mRNA levels in the hypothalamus of adult male zebrafish (135). On the other hand, high temperature results in an increase in *kiss2* transcripts in the head of pejerrey larvae at week 4 after hatching. It is important to note that pejerrey is a fish with strong temperature-dependent sex determination, and high temperatures can result in 100% male offspring. These data suggest that *kiss2* may play an important role in the process of sex differentiation in this species (36).

### Nutritional Status

In mammals, the reproductive axis is known that be regulated by energy balance, and the kisspeptin system appears to play a key role in linking energy balance and reproduction (141). Fasting has been shown to decrease hypothalamic *kiss1* and *kissr1* mRNA levels in mouse and rhesus monkey, *Macaca mulatta* (142, 143). Moreover, fasting in rat results in a concomitant decrease in hypothalamic *kiss1* and an increase in *kissr1* mRNA levels (144).

In teleosts, kisspeptin systems also appear to be associated with nutritional status. For example, in Senegalese sole, 15 days of starvation results in a significant increase in *kiss2* and *kissr2* mRNA levels in the hypothalamus, but no changes are observed for these two genes in the stomach (7). Similarly, two alternative variants for *kissr3* (*kissr3_v1* and *kissr3_v2*) and *kissr2* (*kissr2_v1* and *kissr2_v2*) are identified in pejerrey, and fasting also increases hypothalamic *kiss2* and *kissr2_v1* mRNA levels, whereas *kissr2_v2* shows no expression in the hypothalamus (65). However, food deprivation has no significant effect on the expression levels of *kissr2_v1* and *kissr2_v2* in the testis and habenula of pejerrey compared to the control group (65).

Also, a longer period of food restriction (14 months) results in an increase in mRNA levels of *kiss1*, *kiss2*, *kissr2* and *kissr3* in the hypothalamus of European sea bass (116). Overall, it appears that the neuroendocrine mechanisms mediating the effect of negative energy balance on reproduction may differ between mammals and teleosts. It is noteworthy that kisspeptin reduces appetite in several mammalian species (145–148). However, whether and how kisspeptins are involved in the regulation of food intake and energy balance in teleosts requires further investigation.

### Sex Steroids

Sex steroids, estrogens and androgens, are important for the differential expression of the elements of the kisspeptin systems. For example, in female medaka, the number of *kiss1* neurons in the NVT, but not in the *nucleus preopticus periventricularis* (NPPv), is significantly reduced after ovariectomy (OVX) compared with the sham-operated group, and basal levels are restored after E_2_ treatment (29). In addition, double-labeling *in situ* hybridization showed that estrogen receptor alfa (Erα) is expressed together with *kiss1* in NVT neurons, suggesting that these neurons are involved in the positive feedback regulation of the BPG axis in this species (132). However, the number of NRL *kiss2* neurons is not altered after OVX, and no ERα transcripts are detected in or in close association to the NRL *kiss2* neurons (132). In contrast, in goldfish, the number of *kiss2* neurons in the POA is downregulated after OVX and is restored by E_2_ administration, and *kiss2* neurons in the POA express all three ER types (149).

In OVX orange-spotted grouper, the expression of *kiss2* but not *kiss1* is significantly increased in the brain, and E_2_ substitution could reverse this effect (150). Bioinformatics analysis of the promoter of kisspeptins and kisspeptin receptors in yellowtail kingfish and zebrafish reveals high abundance of several regulatory elements such as AP-1, Sp1, ER, AR and PR (151), suggesting possible regulation of Kiss genes and their receptors by steroids, especially E_2_. It was also demonstrated that E_2_ is able to positively feedback on the expression of *kiss1* and *kiss2* in goldfish through different ERα pathways (152), and similar results are observed in orange-spotted grouper (150). On the other hand, E_2_ treatment causes a significant increase in mRNA expression of *kiss1*, *kiss2*, and *kissr2* in zebrafish brain, but *kissr3* transcript levels are not altered (16). In addition, a positive effect of E_2_ on the expression of *kiss2* but not *kiss1* is observed in the brain of the sapphire devil, *Chrysiptera cyanea* (153) and in the hypothalamus of the Dabry’s sturgeon, *Acipenser dabryanus* (154).

Kisspeptin receptors are also regulated by gonadal steroids in fish. E_2_ also increases expression of the *kissr2* and *kissr3* genes in the sapphire devil brain (155) and European sea bass pituitary cells (84). Hypothalamic *kissr3* but not *kissr2* transcripts are upregulated in Dabry’s sturgeon after E_2_ injection (154), whereas neither *kiss2* nor *kissr2* mRNA levels are altered by E_2_ in the hypothalamus of half-smooth tongue sole (156). Interestingly, no significant changes in hypothalamic *kiss1*, *kiss2* and their receptors mRNA levels are observed in European sea bass by E_2_ treatment after OVX, as determined by qRT-PCR. However, the number of *kiss1* and *kiss2* expressing cells is reduced in some brain regions, and E_2_ replacement prevents this effect, as revealed by *in situ* hybridization (157).

Androgens have also been shown to mediate feedback on the regulation of kisspeptin neurons. Transcript levels of *kiss1*, *kiss2*, and *kissr2* in the brain are reduced by T treatment of OVX female striped bass during mid-vitellogenesis (158). Similarly, T administration reduces mRNA levels of *kiss1*, *kiss2*, and *kissr2* in the brain of gonadectomized (GDX) at mid-gonadal development of male striped bass. In contrast, pubertal males responds to T replacement by up-regulation of *kiss1* and *kiss2*, whereas no changes are observed in juvenile and recrudescent males, suggesting a differential and gonadal stage-dependent role of T in regulating mRNA levels of *kiss1* and *kiss2* (133). On the other hand, a negative feedback effects of T on hypothalamic *kiss2* expression is observed in GDX European sea bass males, without affecting *kiss1*, *kissr2* and *kissr3* mRNA levels (157). However, T has no effect on the expression of the elements of kisspeptin system in the hypothalamus of half-smooth tongue sole (156) and midbrain of goldfish (159). A stimulatory effect of T on mRNA levels of *kissr2* and *kissr3* is detected in primary cultured pituitary cells of European sea bass (84). Taken together, these results suggest that the regulation of genes encoding kisspeptins and their receptor by gonadal steroids in teleosts depends on the species, tissue, gene, reproductive stage, and route of administration and that needs to be investigated in each individual species.

### Neuropeptides Related to Reproduction

In teleosts, negative and positive feedbacks were described for kisspeptins on their own expression. For example, Kiss1 administration decreases the amount of *kiss1* mRNA in the habenula of zebrafish (160) and induces a higher expression of *kissr2* in the brain of fathead minnow (40). Similarly, Kiss2 stimulates *kissr2* mRNA levels in primary cultured brain cells of Japanese flounder (100). Both *kiss2* and *kissr2* transcript levels are significantly increased in the hypothalamus of black porgy, *Acanthopagrus schlegelii*, after injection with Kiss2 (99). In addition, exogenous administration of Kiss2 increases gene expression of reproduction-related genes (*gnrh3*, *kissr2*, *fshb*, *lhb*, *ar*, and *er2*), sex hormone levels (E_2_ and T), and accelerates the onset of puberty in largemouth bass, *Micropterus salmoides* (96). On the other hand, Kiss2 increases hypothalamic *kiss2* expression in half-smooth tongue sole, and decreases *kissr2* mRNA levels (89). In addition, a negative effect of Kiss2 is found on the mRNA abundance of *kissr2_v1* and *kissr2_v5* in the brain of male yellowtail kingfish, while the mRNA levels of *kissr2_v4* are significantly increased (68).

Injection of Kiss2 does not alter *kissr2* mRNA levels in the hypothalamus of lined seahorse, *Hippocampus erectus* (93). Neither Kiss1 nor Kiss2 alters the transcript levels of *kissr2* and *kissr3* mRNAs in the hypothalamus of European sea bass, and *kissr3* mRNA levels in the forebrain-midbrain are not altered by these two peptides (83). However, *kissr2* gene expression is increased in the forebrain-midbrain after exposure to Kiss1 and Kiss2 (83). Interestingly, a differential and gonadal stage-dependent roles of Kiss1 and Kiss2 in regulating *kissr2* expression in hybrid bass brain is observed (17). Transcript levels of *kissr2* are increased only by Kiss2 in prepuberty, whereas a significant decrease in mRNA levels is observed after treatment with Kiss1 and Kiss2 in recrudescence (17).

LPXRFa may also induce differential effects on the expression of kisspeptins and their receptor in teleosts. Intracerebroventricular (icv) injection of LPXRFa-2 suppresses *kiss1*, *kiss2*, and *kissr3* transcripts in the brain of male European sea bass, without affecting *kissr2* expression (161). However, intramuscular (im) injection of LPXRFa-2 significantly increases *kissr3* expression and has no effect on the expression of the other genes (162). On the other hand, no changes in the expression of these four kisspeptin genes (*kiss1*, *kiss2*, *kissr2*, and *kissr3*) are observed after administration of LPXRFa-1 in the same studies (161, 162). In half-smooth tongue sole, neither LPXRFa-1 nor LPXRFa-2 alters hypothalamic *kiss2* mRNA levels *in vitro* (163). Similarly, im injection of LPXRFa-2 and LPXRFa-3 does not alter *kiss2* gene expression in the brain of Senegalese sole (164), and none of the three LPXRFa peptides alters hypothalamic *kiss1* and *kiss2* mRNA levels in orange-spotted grouper (165).

In mammals, kisspeptin is considered an upstream regulator of Gnrh secretion, and although the situation is clearly different in teleosts, Gnrh may exert feedback on gene expression of kisspeptin systems. A mammalian GnRH analog, [D-Ala^6^, Pro^9^Net]-mGnRHa, has a stimulatory effect on the expression of *kiss2* in European sea bass pituitary cell cultures but has no effect on the mRNA levels of *kissr2* and *kissr3* (84). Furthermore, no significant differences in hypothalamic abundance of *kiss2* and *kissr2* mRNAs are observed after exposure to the aforementioned GnRHa in half-smooth tongue sole (89). Similarly, treatment with GnRHa has no effects on the expression levels of *kissr2* in the brain, pituitary gland, and gonads in male yellowtail kingfish (88). Overall, these results suggest a complex control of the kisspeptin system, and each neuropeptide exerts a differential effect on kisspeptin gene regulation, which could depend on the species, sex, tissues, reproductive stages of the animals, peptides used, dose, route of administration, and elapsed time after treatment.

### Other Factors

Thyroid hormones (T_3_ and T_4_) play an important role in the control of growth, morphogenesis, metabolism, and reproduction in several species, including fish (166, 167). Moreover, T_3_ ip administration significantly increases hypothalamic *kiss2* gene in sexually mature male Nile tilapia, whereas this gene is suppressed under a hypothyroid condition induced by methimazole treatment (168).

Endocrine disrupting chemicals (EDCs) can also affect reproductive regulation, in part by affecting kisspeptins system, which is a clear example of neuroendocrine disruption (169). For example, bisphenol-A shows a greatly increased expression of *kiss1*, *kiss2*, and *kissr2* in the brains of pubertal Catla (*Catla catla)* without affecting mRNA levels of *kissr3* (170). In addition, bisphenol-F leads to an increase in the expression of *kiss1* and *kissr3* in the brain of zebrafish but has no effect on the mRNA levels of *kiss2* and *kissr2* (171). In adult male goldfish exposed to vinclozolin, a pesticide that acts as an antiandrogen and impairs reproduction in mammals, *kiss1* but not *kiss2* mRNA levels are increased in the midbrain (159). Similarly, the antiandrogen flutamide also induces *kiss1* and *kiss2* gene expression in the midbrain of goldfish (159). All these data suggest that these EDCs act on steroid receptors and/or steroid balance.

On the other hand, semicarbazide (SMC), an industrially produced synthetic hydrazine compound, significantly downregulates mRNA expression of *kiss2* and *kissr2* in the brain of female Japanese flounder (172). An inhibitory effect of SMC on *kissr2* expression in the brain is also observed in male Japanese flounder (173). Moreover, mRNA levels of *kissr2* and *kissr3* are significantly reduced in the brain of adult female Japanese medaka after chronic exposure to Roundup, a glyphosate-based herbicide. However, neither *kiss1* nor *kiss2* transcripts are altered (174). Moreover, these EDCs may act on the kisspeptins system by mimicking the effects of gonadal steroids, as plasma E_2_ and T levels can be altered by EDCs (93, 173).

Interestingly, other less studied factors, such as social status may also regulate mRNA levels of *kissr* in the entire brain of mouthbrooding cichlids, with higher mRNA levels of *kissr2* observed in high-status territorial males compared to non-territorial males (75).

## Conclusions and Future Directions

In fish, kisspeptins may exert their functions by acting at multiple levels of the brain-pituitary-gonadal axis. Two recent reviews focusing on fish and vertebrates highlighted the different pathways by which kisspeptins may be involved in reproduction, discussed the levels and nature of action, and interaction with Gnrh and other neuropeptides (15, 43). In this review, attention was focused on the whole reproductive brain-pituitary-gonadal axis. Unlike mammals, *kiss/kissr* null zebrafish and medaka can reproduce normally, suggesting that kisspeptin is either not essential for reproduction or that there are compensation mechanisms exerted by other neuropeptides. Teleost are known for their neuroplasticity and multifactorial control of reproduction, with new reproductive neuropeptides emerging (175–177).

With respect to Kiss/Kissr diversity and evolution, we focused particularly on Pleuronectiformes because this order is a good model from an evolutionary perspective and multiple genomes are currently available. Moreover, in Pleuronectiformes, previous studies have mentioned that the kisspeptin-1 system seems to have been lost during evolution (8, 15, 45), but recent synteny and phylogenetic analysis has shown that this is not so clear for all species in this group. In addition, four rounds of genome duplication are known to have occurred in salmonids (178), but no additional *kiss or kissr* have been found to date. Therefore, it will be interesting to search for orthologous pseudogenes in salmonid genomes.

Most studies on kisspeptin in fish have focused on reproduction, while the role of the kisspeptin system in peripheral tissues is still unclear and there are important questions to be addressed. For example, kisspeptin suppresses food intake in some mammalian species, such as mice, rats, and desert jerboas (145–148). Whether and how kisspeptin regulates appetite and energy balance in teleosts is not yet clear and requires further investigation. Further studies are also needed to elucidate the roles of the kisspeptin systems in development, metabolism, and behavior, as well as to explore the intracellular signaling pathways involved in kisspeptin actions and possible interactions with other neuroendocrinological factors in teleosts.

## Author Contributions

BW, ASM, and GMS contributed equally to the manuscript. All authors contributed to the article and approved the submitted version.

## Funding

This study was carried out with financial support from a project funded by the Agencia Nacional de Promoción Científica y Tecnológica (ANPCYT, Argentina) PICT-2015-2783 to GMS. and PICT-2017- 2839 to ASM, the National Natural Science Foundation of China 32072949 and the Laboratory for Marine Fisheries and Food Production Processes, Pilot National Laboratory for Marine Science and Technology (Qingdao) ZZ-B06 to BW, who also received a scholarship supported by the China Scholarship Council (CSC, File No. 201903260004).

## Conflict of Interest

The authors declare that the research was conducted in the absence of any commercial or financial relationships that could be construed as a potential conflict of interest.

## Publisher’s Note

All claims expressed in this article are solely those of the authors and do not necessarily represent those of their affiliated organizations, or those of the publisher, the editors and the reviewers. Any product that may be evaluated in this article, or claim that may be made by its manufacturer, is not guaranteed or endorsed by the publisher.
